# Cloning and Functional Analysis of *ZFP5* from *Amorpha fruticosa* for Enhancing Drought and Saline–Alkali Resistance in Tobacco

**DOI:** 10.3390/ijms26083792

**Published:** 2025-04-17

**Authors:** Ziang Liu, Yu Yang, Lihua Yang, Bochun Wang, Xiaotong Gao, Shuchao Huang, Xiufeng Li, Chengjun Yang, Qingjie Guan

**Affiliations:** 1 Key Laboratory of the Ministry of Education for Ecological Restoration of Saline Vegetation, College of Life Sciences, Northeast Forestry University, Harbin 150040, China; 1783771028@nefu.edu.cn (Z.L.); 2024122963@nefu.edu.cn (Y.Y.); 18315847959@nefu.edu.cn (L.Y.); 2024113068@nefu.edu.cn (X.G.); huangshuchao@nefu.edu.cn (S.H.); 2 Aulin College, Northeast Forestry University, Harbin 150040, China; 3264496926@nefu.edu.cn; 3 Key Laboratory of Soybean Molecular Design Breeding, Northeast Institute of Geography and Agroecology, Chinese Academy of Sciences, Harbin 150081, China; lixiufeng@iga.ac.cn; 4 College of Forestry, Northeast Forestry University, Harbin 150040, China

**Keywords:** abiotic stress, *Amorpha fruticosa*, zinc finger protein, tobacco, genetic transformation

## Abstract

Drought and soil salinization significantly constrain agricultural productivity, driving the need for molecular breeding strategies to enhance stress resistance. Zinc finger proteins play a critical role in plant response to abiotic stress. In this study, a gene encoding a C2H2-type zinc finger protein (*AfZFP5*) was cloned from *Amorpha fruticosa,* a species known for its strong adaptability. qRT-PCR analysis revealed that *AfZFP5* expression is regulated by sorbitol, H_2_O_2_, NaCl, and NaHCO_3_. And all four treatments can cause upregulation of *AFZFP5* expression in the roots or leaves of *Amorpha fruticosa* within 48 h. Transgenic tobacco lines overexpressing AfZFP5 demonstrated enhanced tolerance to drought and salt–alkali stress at germination, seedling, and vegetative stages. Compared to wild-type plants, transgenic lines exhibited significantly higher germination rates, root lengths, and fresh weights when treated with sorbitol, NaCl, and NaHCO_3_. Under natural drought and salt–alkali stress conditions, transgenic plants showed elevated activities of superoxide dismutase (SOD) and peroxidase (POD), and upregulated expression of oxidative stress-related kinase genes (NtSOD, NtPOD) during the vegetative stage. Additionally, transgenic tobacco displayed lower malondialdehyde (MDA) content and reduced staining levels with 3,3′diaminobenzidine (DAB) and Nitro blue tetrazolium (NBT), indicating enhanced reactive oxygen species (ROS) scavenging capacity by *AfZFP5* upon salt–alkali stress. Under simulated drought with PEG6000 and salt–alkali stress, chlorophyll fluorescence intensity and Fv/Fm values in transgenic tobacco were significantly higher than in wild-type plants during the vegetative stage, suggesting that *AfZFP5* mitigates stress-induced damage to the photosynthetic system. This study highlights the role of *AfZFP5* in conferring drought and salt–alkali stress tolerance, providing genetic resources and a theoretical foundation for breeding stress-resistance crops.

## 1. Introduction

Plants encounter various environmental stresses throughout their entire life cycle which can disrupt normal physiological activities. Over time, natural selection and evolution have enabled plants to develop defense mechanisms to withstand adverse conditions [1]. Upon perceiving stress signals, plants activate signal transduction pathways, triggering molecular and cellular responses, leading to physiological and biochemical adaptations to mitigate stress-induced damage [2,3,4]. Abiotic stress often induces excessive production of reactive oxygen species (ROS), resulting in oxidative damage [5]. To counteract this, plants activate antioxidant defense systems, including enzymes such as superoxide dismutase (SOD), peroxidase (POD), catalase (CAT), and ascorbate peroxidase (APX), along with non-enzymatic antioxidants like anthocyanins, to scavenge excess ROS [6,7,8]. Meanwhile, transcription factors (TFs), such as AP2/ERF, bHLH, bZIP, MYB, NAC, WRKY, and zinc finger proteins (ZFPs), play crucial roles in plant responses to abiotic stress [9,10,11,12,13,14,15]. ZFPs are a superfamily of transcription factors that control numerous activities in plants including growth, development, and cellular responses to biotic and abiotic stresses. ZFPs contain zinc-binding domains that form characteristic finger-like structures. Based on the arrangement of cysteine and histidine residues, ZFPs are classified into various types, including C2H2, C4, C6, C4HC3, C3HC4, C2HC, and C3H [16]. Numerous studies have indicated that ZFPs regulate plant growth, development, disease resistance, and stress responses. Among them, C2H2-type ZFPs have been extensively studied for their role in abiotic stress adaptation [17,18,19]. For instance, PtrZAT12 is induced by cold stress, while overexpression of *PtrZAT10* enhances cold tolerance in transgenic tobacco (*Nicotiana tabacum*). Knocking out *PtrZAT11* in *Poncirus trifoliata L. Raf.* increases cold resistance by suppressing stress-related gene expression [20]. Similarly, heterologous expression of *MpZFP1* in *Arabidopsis* enhances seed germination, seedling survival, and biomass accumulation under salt stress by effectively promoting ROS scavenging [21]. *IbZFP1,* a positive regulator of drought and salt tolerance in sweet potato, enhances stress resilience in transgenic *Arabidopsis* by modulating the ABA signaling pathway and proline biosynthesis [22].

*Amorpha fruticosa* is a perennial woody plant in the legume family, valued for its medicinal, nutritional, and ecological benefits. Its leaves and fruits possess anti-inflammatory and analgesic properties, making them useful in traditional Chinese medicine [23,24]. Additionally, *Amorpha fruticosa* is rich in essential amino acids, making it a high-quality feed resource [25]. Beyond its agricultural and medicinal applications, *Amorpha fruticosa* holds significant ecological value. It plays a key role in soil and water conservation, and is widely used for land greening and forest stabilization [26]. More importantly, *Amorpha fruticosa* exhibits remarkable adaptability to abiotic stress, thriving in harsh environments such as cold, drought, and saline–alkali soils, particularly in Northeast China [27,28].

Given the high application value of *Amorpha fruticosa*, this study aimed to characterize its drought stress response by cloning a ZFP gene, *AfZFP5*, through RNA-seq analysis. The functional role of *AfZFP5* was investigated by subjecting transgenic tobacco plants to drought and salt–alkali stress at different growth stages. By examining phenotypic changes and analyzing key physiological indicators, this study explored the regulatory role of *AfZFP5* in plant stress resistance, seeking a clue for gene editing-based breeding strategies.

## 2. Results

### 2.1. Cloning and Bioinformatics Analysis of the AfZFP5

**Figure 1:**
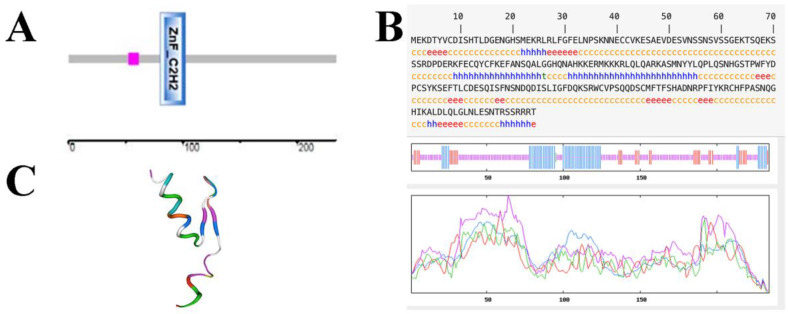
Bioinformatics analysis of AfZFP5. (**A**) The primary structure of the AfZFP5 protein from SMART. (**B**) The predicted secondary structure of the AfZFP5 protein from SWISS-MODEL. (**C**) The predicted tertiary structure of the AfZFP5 protein from SWISS-MODEL.

**Figure 2:**
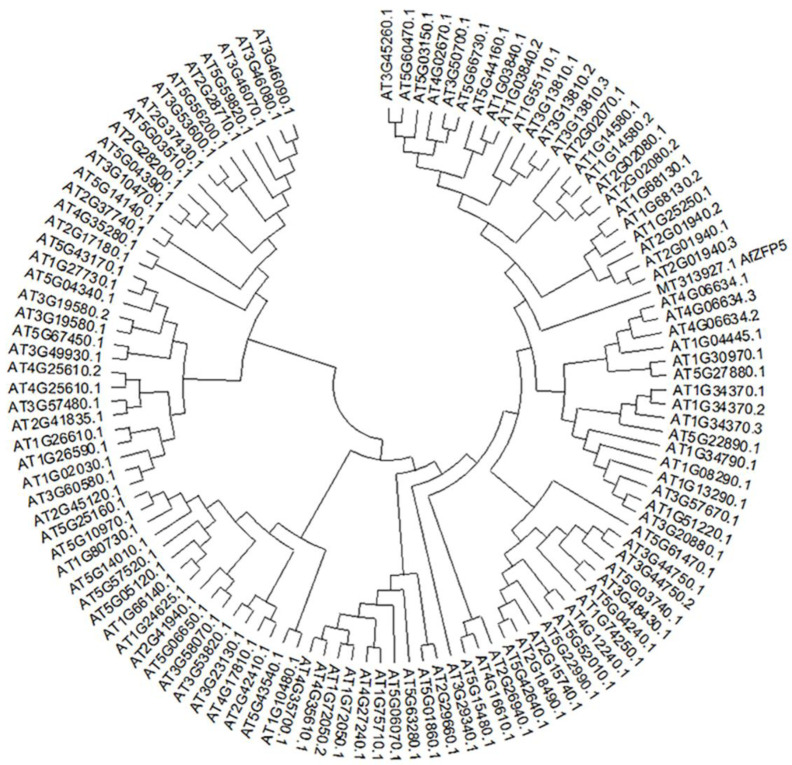
Phylogenetic analysis of AfZFP5.

The *AfZFP5* gene was successfully cloned, NCBI and online software The SMART (SMART: Main page (embl-heidelberg.de)) analysis confirmed that it contains a ZnF-C2H2 domain (Figure 1A). Structural predications using SWISS-MODEL revealed that the AfZFP5 protein comprises four alpha-helix regions, seven extended chain regions, and thirteen randomly coiled regions (Figure 1B,C). Phylogenetic tree analysis demonstrates that AfZFP5 is functionally conserved within the Arabidopsis C2H2 ZFP family (Figure 2).

### 2.2. Analysis of AfZFP5 Expression Characteristics

**Figure 3:**
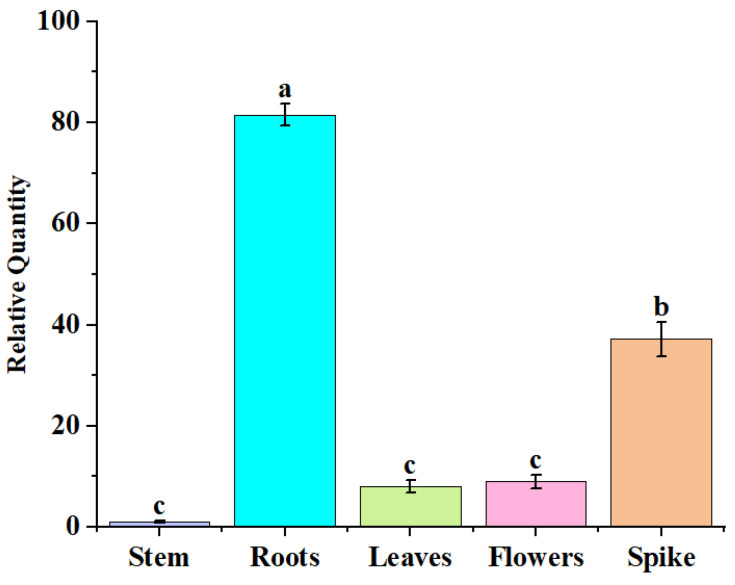
The expression levels of *AfZFP5* in different tissues and organs. Note: The error bars represent the standard errors of three biological replicates. Significant differences were determined at *p* < 0.05. Lowercase letters (a, b, c, etc.) denote statistically significant differences (*p* < 0.05).

qRT-PCR analysis revealed that *AfZFP5* was expressed in different tissues of *Amorpha fruticosa* including roots, stems, leaves, and flowers. The highest expression level was observed in roots, while the lowest expression level was detected in stems (Figure 3).

**Figure 4:**
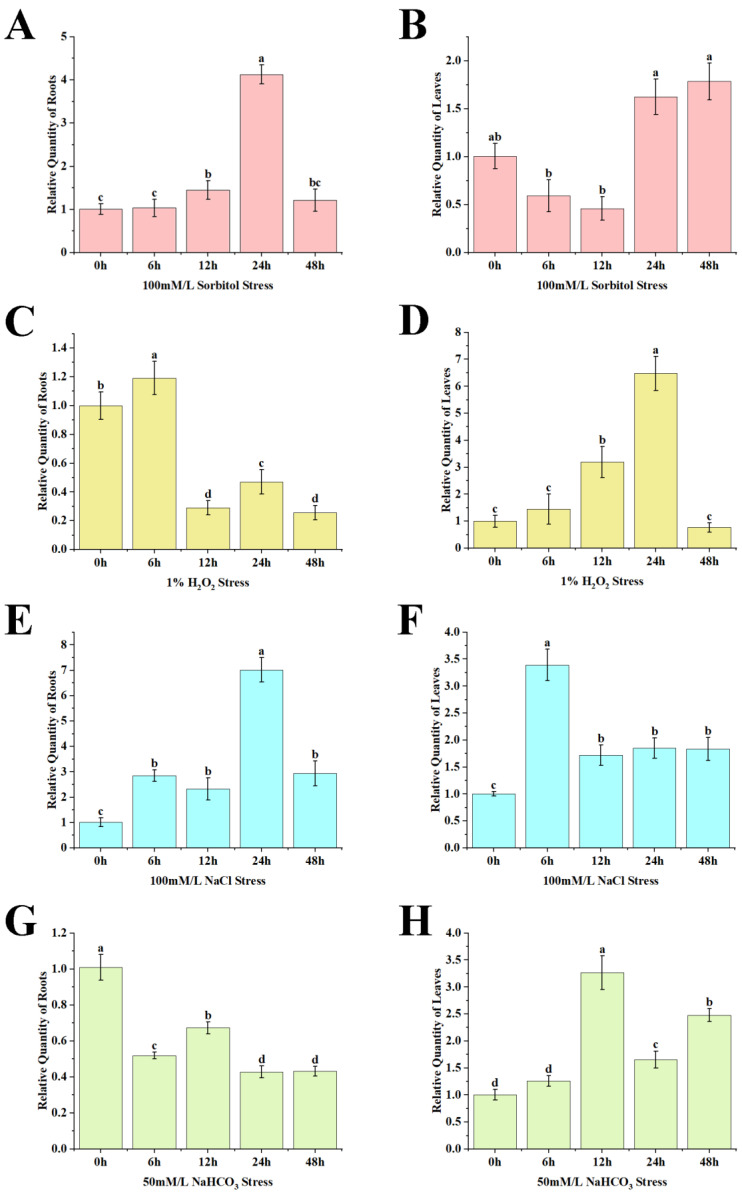
The expression characteristics of *AfZFP5* in *Amorpha fruticosa*. The expression of *AfZFP5* in the (**A**) roots under sorbitol treatment; (**B**) in the leaves under sorbitol treatment; (**C**) in the roots under H_2_O_2_ treatment; (**D**) in the leaves under H_2_O_2_ treatment; (**E**) in the roots under NaCl treatment; (**F**) in the leaves under NaCl treatment; (**G**) in the roots under NaHCO_3_ treatment; and (**H**) in the leaves under NaHCO_3_ treatment. Note: The error bars represent the standard errors of three biological replicates. Significant differences were determined at *p* < 0.05. Lowercase letters (a, b, c, etc.) denote statistically significant differences (*p* < 0.05).

We also examined the differential expression of *AfZFP5* under various treatments, including sorbitol, H_2_O_2_, NaCl, and NaHCO_3_. Under 100 mM sorbitol stress, the expression level of *AfZFP5* in the roots of *Amorpha fruticosa* gradually decreased, reaching its lowest point at 12 h. Afterwards, the expression level stabilized. In contrast, the expression level of *AfZFP5* in the leaves showed a pattern of first decreasing and then increasing. It reached its lowest point at 6 h, followed by a gradual increase, with significantly higher expression levels at 24 h and 48 h compared to the 0 h baseline (Figure 4A,B).

Under 1% H_2_O_2_ stress, the expression level of *AfZFP5* in the roots of *Amorpha fruticosa* significantly decreased at 12 h, after which it remained relatively stable as the stress duration increased. In contrast, the expression level of *AfZFP5* in the leaves showed an initial increase, with a significant rise observed at 24 h of stress treatment and then a significant decrease as the stress treatment continued (Figure 4C,D).

Under 100 mM NaCl stress, the expression level of *AfZFP5* in the roots of *Amorpha fruticosa* was significantly increased, reaching its peak at 24 h. After 48 h, the expression level began to decrease, but remained significantly higher than that at the 0-h baseline. In the leaves, the expression level of *AfZFP5* significantly increased at 6 h, followed by a decrease and a trend toward stabilization as the stress duration extended (Figure 4E,F).

Under 50 mM NaHCO_3_ stress, the expression level of *AfZFP5* in the roots of *Amorpha fruticosa* showed a significant decrease, stabilizing after 24 h of stress. In contrast, *AfZFP5* in the leaves was upregulated, with a significant increase observed at 12 h. Following this peak, it decreased and stabilized as the stress duration continued (Figure 4G,H).

### 2.3. Overexpression of AfZFP5 in Tobacco

To assess whether *AfZFP5* enhances plant defense against abiotic stress, we generated transgenic tobacco lines. Six independent transgenic lines were obtained by transforming the *AfZFP5*-GFP fusion gene driven by the 35S promoter into tobacco. qRT-PCR analysis revealed that strain 3, strain 4, and strain 5 exhibited relatively high expression levels of *AfZFP5*. These three strains were selected for further resistance screening and subsequent analysis (Supplementary Figure S1).

### 2.4. Subcellular Localization of AfZFP5 Gene-Encoded Protein

**Figure 5:**
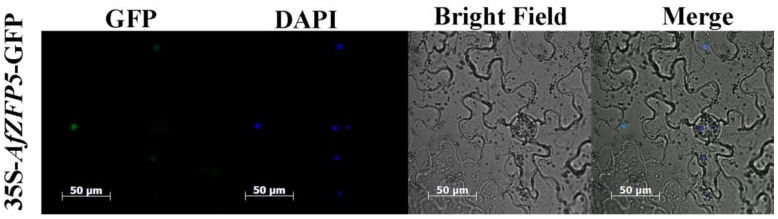
Subcellular localization of AfZFP5 in tobacco cells. Scale bar: 50 μm.

To investigate the subcellular localization of the protein encoded by *AfZFP5*, the Agrobacterium-mediated transient expression system was used to express the AfZFP5-GFP fusion protein in tobacco leaves, driven by the 35S promoter. Microscopic observation revealed that the green fluorescence signal was primarily located in the nucleus, overlapping with the signal of the nucleus-specific dye DAPI. This finding indicates that AfZFP5 is localized to the nucleus and likely functions within this cellular compartment (Figure 5).

### 2.5. Tolerance of AfZFP5 Transgenic Tobacco to Drought and Salt–Alkali Stress During Germination

**Figure 6:**
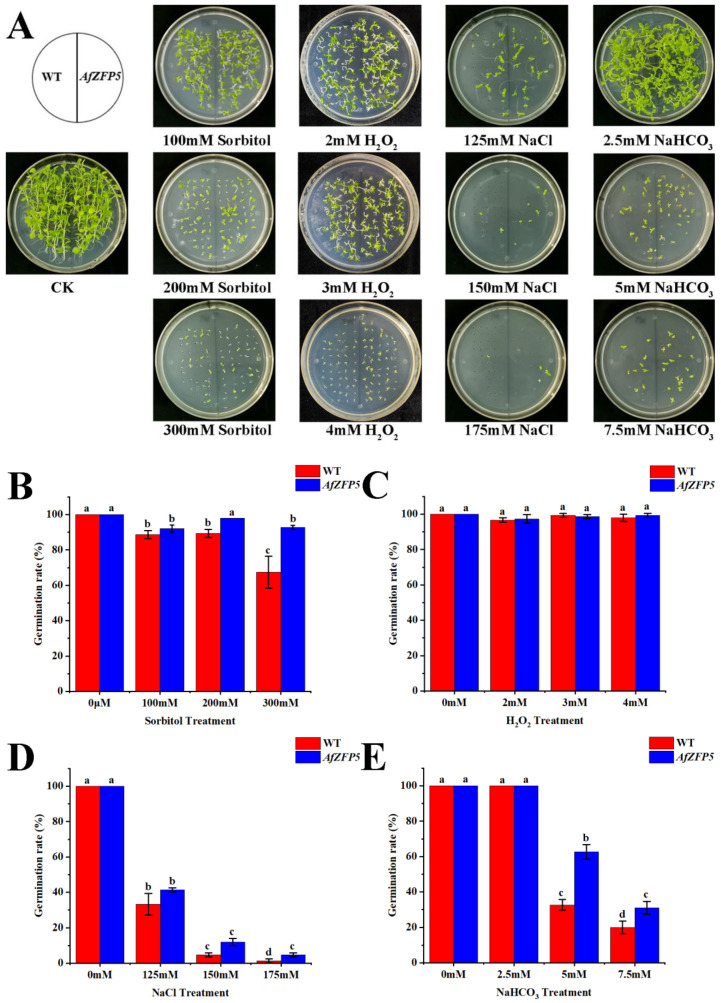
Analysis of T3-5 strain *AfZFP5* transgenic tobacco seed tolerance during germination under sorbitol, H_2_O_2_, NaCl, and NaHCO_3_ treatments. (**A**) The germination phenotype of *AfZFP5* transgenic tobacco seeds under sorbitol, H_2_O_2_, NaCl, and NaHCO_3_ treatments. The germination rate of *AfZFP5* transgenic tobacco seeds under (**B**) sorbitol; (**C**) H_2_O_2_; (**D**) NaCl; and (**E**) NaHCO_3_ treatment. Note: The error bars represent the standard errors of three biological replicates, with significant differences determined at the *p* < 0.05 level. Lowercase letters (a, b, c, etc.) denote statistically significant differences (*p* < 0.05).

This study analyzed the germination rate of *AfZFP5* transgenic tobacco under stress conditions induced by sorbitol, H_2_O_2_, NaCl, and NaHCO_3_. By day 16, the growth status of wild-type tobacco and *AfZFP5* transgenic tobacco was similar under control conditions. However, sorbitol, NaCl, and NaHCO_3_ stress treatments significantly inhibited germination and growth in both wild-type and *AfZFP5* transgenic tobacco, with *AfZFP5* transgenic lines exhibiting greater tolerance to these treatments. H_2_O_2_ stress treatment did not notably influence the germination of either wild-type or *AfZFP5* transgenic tobacco. However, as H_2_O_2_ concentration increased, growth inhibition became gradually obvious in both groups. This suggests that while H_2_O_2_ stress does not significantly hinder germination, it progressively suppresses seedling growth (Figure 6).

### 2.6. Tolerance of AfZFP5 Transgenic Tobacco to Drought and Salt–Alkali Stress During Seedling Stage

**Figure 7:**
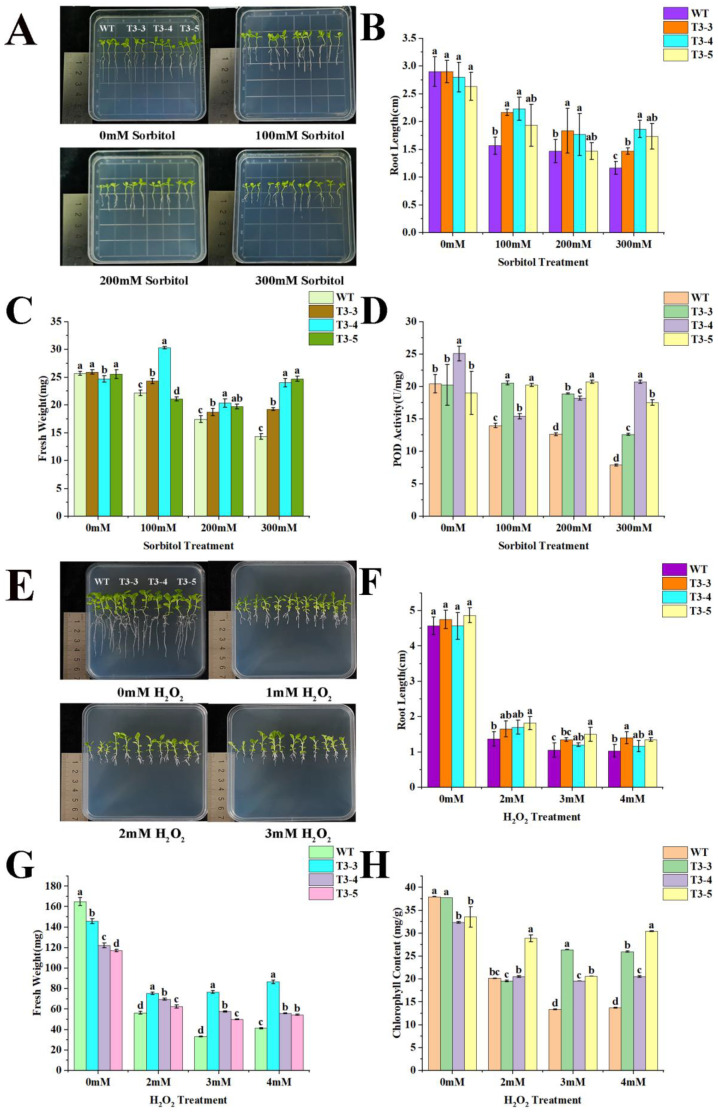

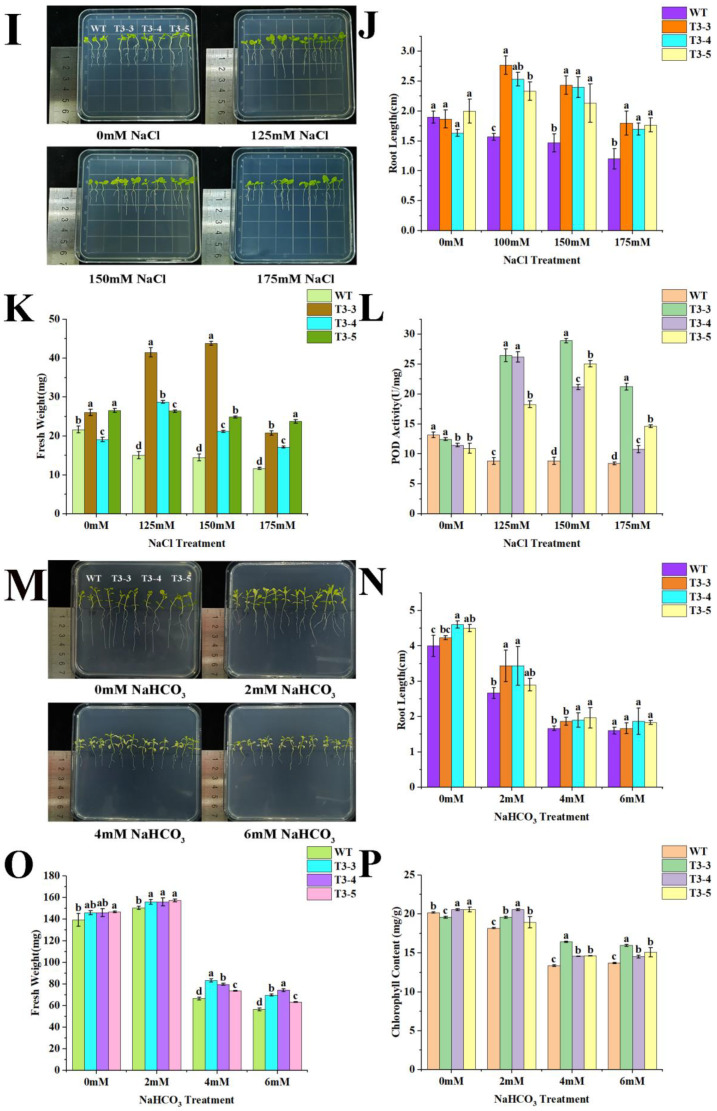
Tolerance analysis of *AfZFP5* transgenic tobacco seedlings under sorbitol, H_2_O_2_, NaCl, and NaHCO_3_ treatments. (**A**) Phenotype under sorbitol stress. (**B**) Root length measurements under sorbitol stress. (**C**) Fresh weight under sorbitol stress. (**D**) POD enzyme activity under sorbitol stress. (**E**) Phenotype under H_2_O_2_ stress. (**F**) Root length under H_2_O_2_ stress. (**G**) Fresh weight under H_2_O_2_ stress. (**H**) Chlorophyll content under H_2_O_2_ stress. (**I**) Phenotype under NaCl treatment. (**J**) Root length under NaCl treatment. (**K**) Fresh weight under NaCl treatment. (**L**) POD enzyme activity under NaCl treatment. (**M**) Phenotype under NaHCO_3_ treatment. (**N**) Root length under NaHCO_3_ treatment. (**O**) Fresh weight under NaHCO_3_ treatment. (**P**) Chlorophyll content under NaHCO_3_ treatment. Note: Error bars represent standard errors of three biological replicates, with significant differences determined at *p* < 0.05 level. Lowercase letters (a, b, c, etc.) denote statistically significant differences (*p* < 0.05).

To assess the tolerance of *AfZFP5* transgenic tobacco to drought and salt–alkali stress at the seedling stage, seedlings were cultivated for 10 days in 1/2 MS medium supplemented with 100 mM, 200 mM, and 300 mM sorbitol. In the control group (1/2 MS medium without stress treatment), the growth of wild-type tobacco and the three *AfZFP5* transgenic lines was comparable, with no significant difference in root length and fresh weight. Under sorbitol stress, both wild-type tobacco and *AfZFP5* transgenic tobacco exhibited inhibited growth, with increasing sorbitol concentrations exacerbating the effect. However, the three *AfZFP5* transgenic lines maintained significantly greater root length and fresh weight compared to wild-type tobacco. Additionally, POD activity measurements revealed that under sorbitol stress, the POD activity of *AfZFP5* transgenic tobacco was significantly higher than that of wild-type tobacco. These results suggest that *AfZFP5* enhances tobacco’s tolerance to sorbitol-induced drought stress (Figure 7A–D).

To evaluate the tolerance of *AfZFP5* transgenic tobacco to oxidative stress at the seedling stage, seedlings were cultivated for 16 days in 1/2 MS medium supplemented with 2 mM, 3 mM, and 4 mM H_2_O_2_. In the control group (1/2 MS medium without stress treatment), the growth of wild-type tobacco and the three *AfZFP5* transgenic tobacco strains was similar, with no significant difference in root length. Under H_2_O_2_ stress, both wild-type and *AfZFP5* transgenic tobacco exhibited inhibited growth, with significantly reductions in root length, fresh weight, and chlorophyll content. However, compared to wild-type tobacco, *AfZFP5* transgenic tobacco maintained significantly greater root length, fresh weight, and chlorophyll content under H_2_O_2_ stress. These findings suggest that *AfZFP5* enhances tobacco’s tolerance to oxidative stress (Figure 7E–H).

To assess the impact of *AfZFP5* on salt stress tolerance, *AfZFP5* transgenic and wild-type tobacco seedlings were cultivated for 7 days in 1/2 MS medium supplemented with 125 mM, 150 mM, and 175 mM NaCl. In the control group (1/2 MS medium without stress treatment), the growth of wild-type and transgenic tobacco was comparable, with no significant difference in root length. Upon NaCl stress, the root length and fresh weight of wild-type tobacco were significantly inhibited. However, 125 mM and 150 mM NaCl stress promoted root elongation in the three *AfZFP5* transgenic lines, with the most pronounced effect observed under 125 mM NaCl. At 175 mM NaCl, the root length of *AfZFP5* transgenic tobacco returned to control levels, with no observed inhibitory effects. Additionally, 125 mM and 150 mM NaCl significantly increased the fresh weight of the *AfZFP5* transgenic T3-3 strain. Furthermore, under NaCl stress, POD activity decreased in wild-type tobacco, but significantly increased in AfZFP5 transgenic lines, surpassing wild-type levels. These results suggest that *AfZFP5* enhances tobacco’s tolerance to NaCl stress (Figure 7I–L).

To evaluate the role of *AfZFP5* in alkali stress tolerance, *AfZFP5* transgenic and wild-type tobacco seedlings were cultivated for 14 days in 1/2 MS medium supplemented with 2 mM, 4 mM, and 6 mM NaHCO_3_. Growth inhibition was observed in both wild-type and *AfZFP5* transgenic tobacco under NaHCO_3_ stress, with the severity increasing at higher concentrations. Under 2 mM and 4 mM NaHCO_3_, two *AfZFP5* transgenic lines showed significantly longer root lengths compared to wild-type tobacco. However, at 6 mM NaHCO_3_, root lengths of both wild-type and transgenic tobacco became comparable. Fresh weight was promoted under 2 mM NaHCO_3_ but was inhibited at 4 mM and 6 mM. Notably, despite the stress, the fresh weight of *AfZFP5* transgenic tobacco remained significantly higher than that of wild-type tobacco. Additionally, chlorophyll content measurement showed that *AfZFP5* transgenic tobacco maintained significantly higher chlorophyll levels compared to wild-type tobacco under NaHCO_3_ stress. These results suggest that *AfZFP5* enhances tobacco’s tolerance to NaHCO_3_ stress (Figure 7M–P).

### 2.7. Tolerance of AfZFP5 Transgenic Tobacco to Drought

**Figure 8:**
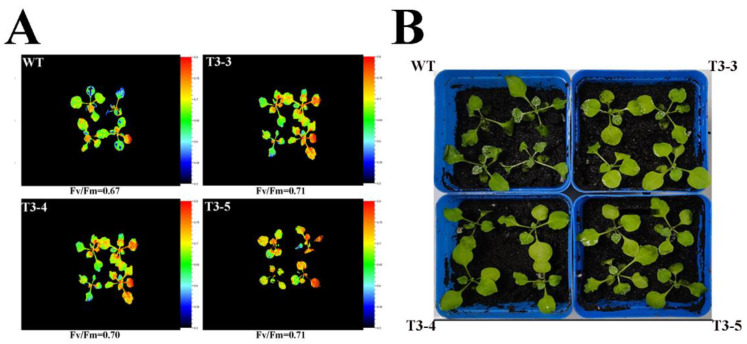
Drought tolerance analysis of *AfZFP5* transgenic tobacco under PEG6000- simulated stress. (**A**) Chlorophyll fluorescence imaging of *AfZFP5* transgenic tobacco during the vegetative stage under PEG6000-simulated drought treatment. (**B**) The phenotype of *AfZFP5* transgenic tobacco during the vegetative stage under PEG6000-simulated drought treatment.

On the third day of PEG6000-simulated drought treatment, the chlorophyll fluorescence intensity of *AfZFP5* transgenic tobacco was stronger than that of wild-type tobacco. Chlorophyll plays a key role in maintaining photosynthesis and serves as an indicator of plant’s tolerance to stress. The changes in Fv/Fm parameters before and after treatments indicate that *AfZFP5* transgenic tobacco exhibits greater drought tolerance than wild-type tobacco (Figure 8A). By day 7, wild-type tobacco leaves showed significant wilting, whereas *AfZFP5* transgenic tobacco leaves exhibited only mild curling, further supporting the enhanced drought resistance of *AfZFP5* transgenic tobacco compared to wild-type tobacco (Figure 8B).

**Figure 9:**
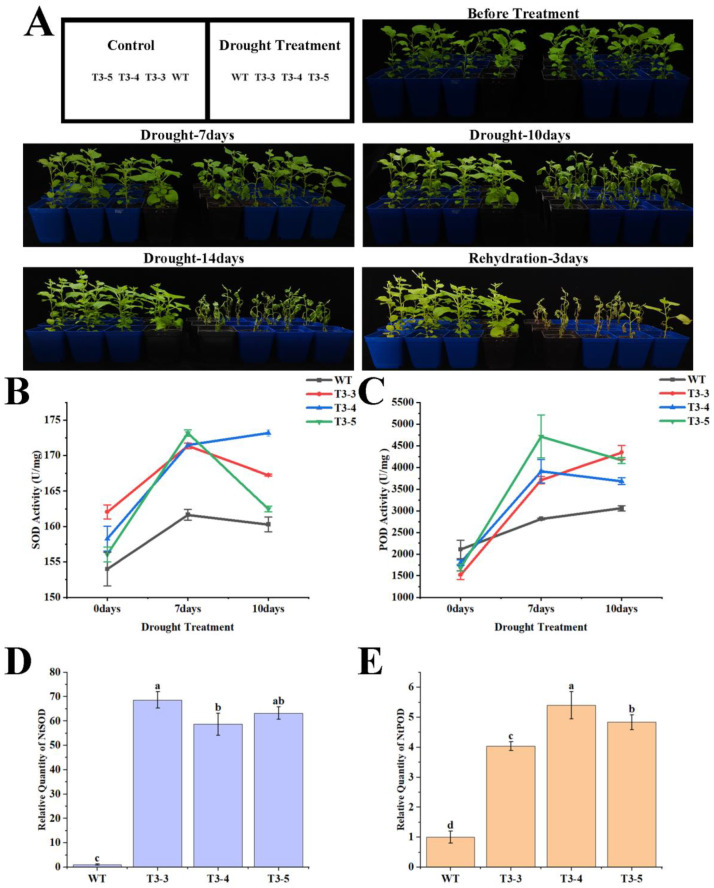
The tolerance of *AfZFP5* transgenic tobacco to natural drought stress during the nutritional stage. (**A**) The phenotype under natural drought treatment. (**B**) SOD enzyme activity under natural drought treatment. (**C**) POD enzyme activity under natural drought treatment. (**D**) The relative expression levels of SOD marker genes under natural drought treatment. (**E**) The relative expression levels of POD marker genes under natural drought treatment. Note: The error bars represent the standard errors of three biological replicates, with significant differences determined at the *p* < 0.05 level. Lowercase letters (a, b, c, etc.) denote statistically significant differences (*p* < 0.05).

After 7 days of natural drought treatment, both wild-type and *AfZFP5* transgenic tobacco exhibited slight wilting, but *AfZFP5* transgenic plants continued to grow normally. Compared to pre-treatment conditions, *AfZFP5* transgenic tobacco plants were significantly taller than wild-type ones. After 10 days of drought stress, wilting became more pronounced in both groups, but *AfZFP5* transgenic tobacco exhibited milder symptoms. By day 14, wild-type tobacco was near complete withering, while *AfZFP5* transgenic tobacco showed severe wilting. Following 3 days of rehydration, only two strains of *AfZFP5* transgenic tobacco, T3-4 and T3-5, began to recover, with a recovery rate of 22.2%. These results further indicate that *AfZFP5* enhances drought resistance (Figure 9A).

On the 7th day of natural drought treatment, both SOD and POD activities significantly increased, with *AfZFP5* transgenic tobacco exhibiting significantly higher levels than wild-type tobacco. SOD and POD activities reflect a plant’s ability to neutralize oxygen free radicals, which plays a crucial role in defending against drought-induced oxidative stress. *AfZFP5* transgenic tobacco had stronger regulatory capacity, suggesting greater drought resistance. As drought stress continued, SOD and POD activities either plateaued or declined by the 10th day, likely due to excessive stress impairing normal physiological functions (Figure 9B,C). Following natural drought treatment, *AfZFP5* transgenic tobacco showed significant upregulation of SOD and POD marker genes compared to wild-type tobacco. SOD gene expression was upregulated by approximately 60-fold, while POD gene expression was upregulated by about 5-fold. This indicates that under drought stress treatment, the enhanced SOD and POD activities in *AfZFP5* transgenic tobacco may be attributed to the elevated expression of their respective marker genes (Figure 9D,E).

### 2.8. Tolerance of AfZFP5 Transgenic Tobacco to Salt–Alkali Stress

**Figure 10:**
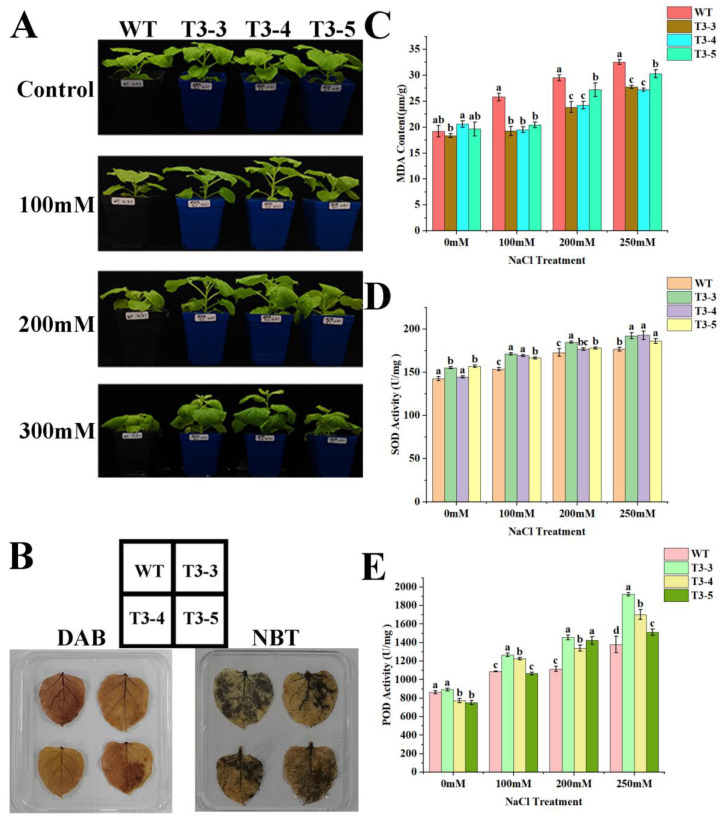
Tolerance analysis of *AfZFP5* transgenic tobacco during the vegetative stage under NaCl stress. (**A**) The phenotype under NaCl stress. (**B**) DAB and NBT staining under NaCl stress. (**C**) The MDA content under NaCl stress. (**D**) SOD enzyme activity under NaCl stress. (**E**) POD enzyme activity under NaCl stress. Note: The error bars represent the standard errors of three biological replicates, with significant differences determined at the *p* < 0.05 level. Lowercase letters (a, b, c, etc.) denote statistically significant differences (*p* < 0.05).

NaCl treatment inhibited tobacco growth, with the inhibitory effect becoming more pronounced as NaCl concentration increased. At a concentration of 200 mM NaCl, tobacco leaves exhibited curling, and the degree of curling intensified with higher NaCl concentrations. However, the growth of *AfZFP5* transgenic strains (T3-3 and T3-4) was significantly better than that of wild-type tobacco. These results indicate that *AfZFP5* transgenic tobacco exhibited stronger tolerance to NaCl stress compared to wild-type tobacco (Figure 10A). The accumulation of H_2_O_2_ and superoxide anions, detected through DAB and NBT staining, reflects the extent of stress-induced damage in tobacco. After staining, wild-type tobacco leaves displayed darker coloration compared to *AfZFP5* transgenic tobacco leaves, indicating that wild-type tobacco experienced greater stress-induced damage (Figure 10B). MDA, a product of membrane lipid peroxidation, serves as an indicator of cellular damage under stress. Higher MDA content corresponds to more severe stress-induced damage. Under NaCl stress, MDA content in both wild-type tobacco and *AfZFP5* transgenic tobacco increased with rising NaCl concentrations. However, at all tested NaCl concentrations, wild-type tobacco exhibited significantly higher MDA content than *AfZFP5* transgenic tobacco, indicating that *AfZFP5* enhanced tolerance to NaCl stress (Figure 10C). Additionally, *AfZFP5* transgenic tobacco showed higher activities of SOD and POD than wild-type tobacco, further supporting its role in conferring stronger tolerance to NaCl stress (Figure 10D,E).

**Figure 11:**
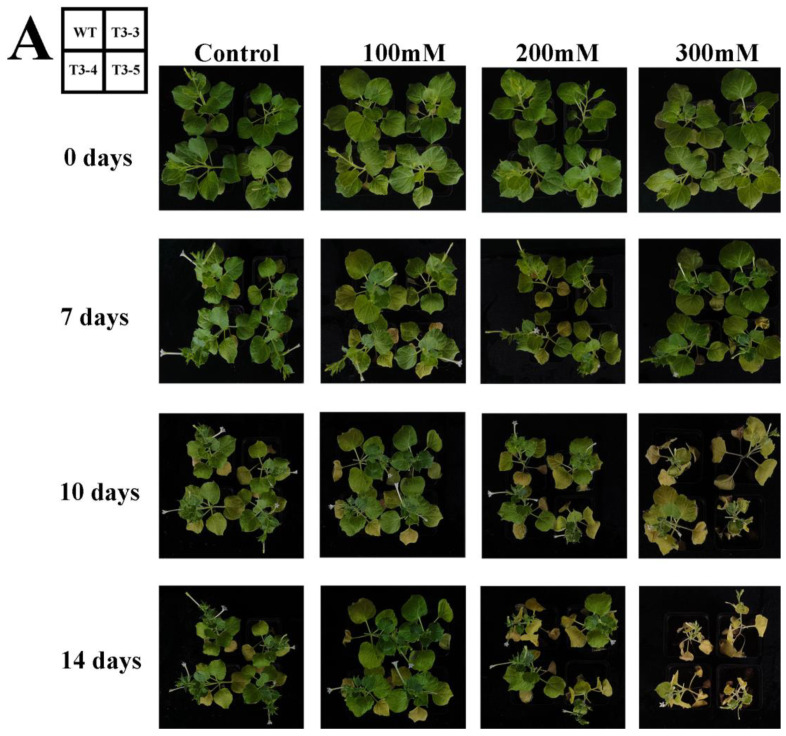

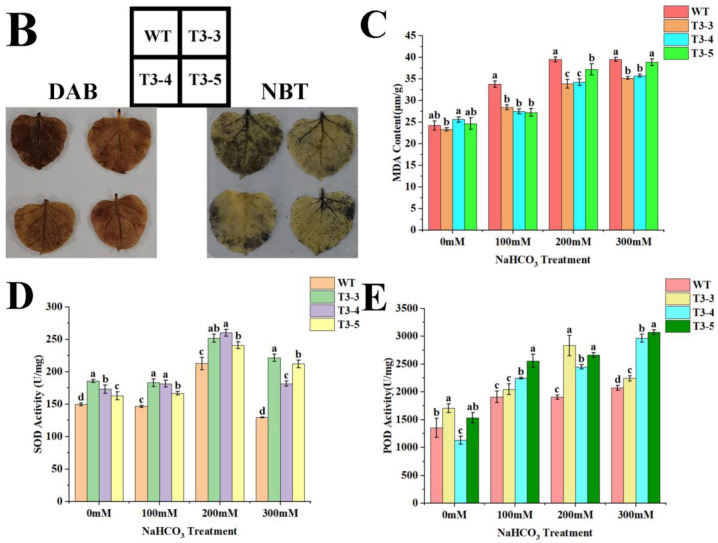
Tolerance analysis of *AfZFP5* transgenic tobacco during the vegetative stage under NaHCO_3_ stress. (**A**) The phenotype under NaHCO_3_ stress. (**B**) DAB and NBT staining under NaHCO_3_ stress. (**C**) The MDA content under NaHCO_3_ stress. (**D**) SOD enzyme activity under NaHCO_3_ stress. (**E**) POD enzyme activity under NaHCO_3_ stress. Note: The error bars represent the standard errors of three biological replicates, with significant differences determined at the *p* < 0.05 level. Lowercase letters (a, b, c, etc.) denote statistically significant differences (*p* < 0.05).

After 7 days of NaHCO_3_ treatment, tobacco leaves exhibited yellowing and curling. At 10 days of treatment, the degree of leaf curling worsened, but the growth of *AfZFP5* transgenic (T3-3 and T3-4) strains under 300 mM NaHCO_3_ was significantly better than that of wild-type tobacco. At 14 days of treatment, wild-type tobacco showed severe wilting under 200 mM NaHCO_3_ treatment, while the T3-3 and T3-4 strains of *AfZFP5* transgenic tobacco remained relatively healthy. Under 300 mM NaHCO_3_ treatment, wild-type tobacco died, whereas *AfZFP5* transgenic tobacco survived. These results indicate that *AfZFP5* enhances tolerance to NaHCO_3_ stress (Figure 11A). DAB and NBT staining revealed that wild-type tobacco leaves displayed darker coloration than *AfZFP5* transgenic tobacco leaves. Additionally, under all three NaHCO_3_ concentrations tested, the MDA content in wild-type tobacco was significantly higher than that in *AfZFP5* transgenic tobacco, indicating that NaHCO_3_ stress caused more severe damage to wild-type tobacco (Figure 11B,C). SOD activity initially increased but then decreased with rising NaHCO_3_ concentrations. This decline may be due to excessive stress damage caused by prolonged exposure to 300 mM NaHCO_3_, which likely inhibited SOD synthesis. However, at all time-points and concentrations, *AfZFP5* transgenic tobacco maintained higher SOD activity compared to wild-type tobacco. Similarly, POD activity gradually increased with higher NaHCO_3_ concentrations, but *AfZFP5* transgenic tobacco consistently exhibited higher POD activity compared to wild-type tobacco. These results further demonstrate that *AfZFP5* enhances tolerance to NaHCO_3_ stress (Figure 11D,E).

**Figure 12:**
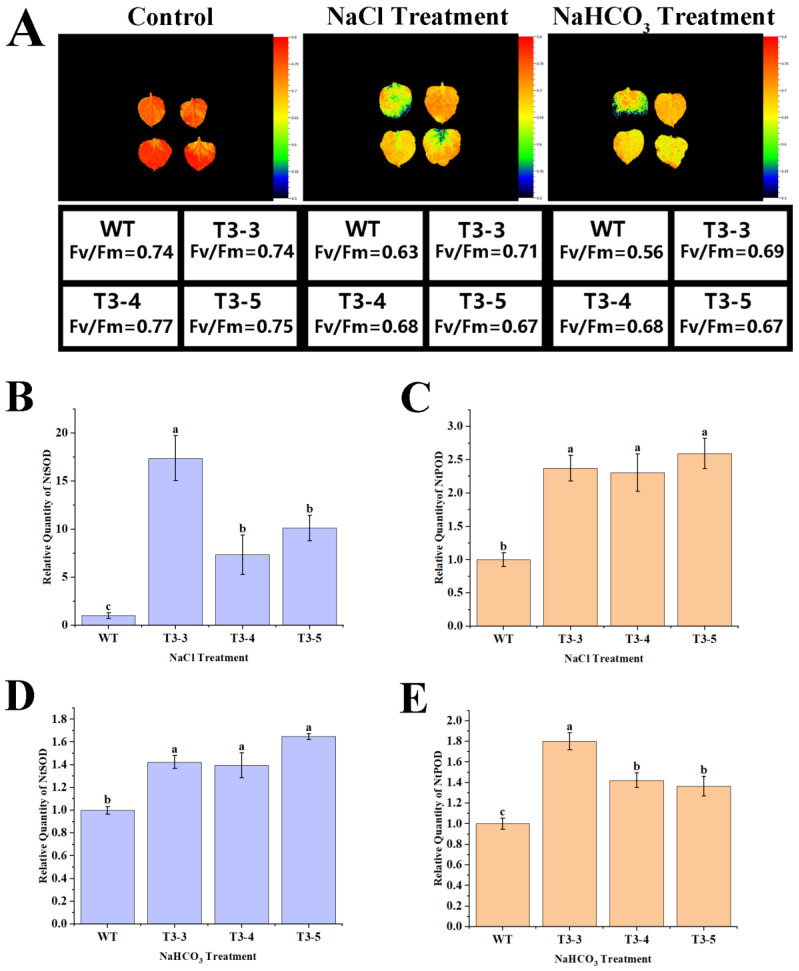
Physiological change analysis of *AfZFP5* transgenic tobacco during the vegetative stage under salt–alkali stress. (**A**) Chlorophyll fluorescence under salt–alkali stress. (**B**) The relative expression levels of SOD enzyme marker genes under NaCl stress. (**C**) The relative expression levels of POD enzyme marker genes under NaCl stress. (**D**) The relative expression levels of SOD enzyme marker genes under NaHCO_3_ stress. (**E**) The relative expression levels of POD enzyme marker genes under NaHCO_3_ stress. Note: The error bars represent the standard errors of three biological replicates, with significant differences determined at the *p* < 0.05 level. Lowercase letters (a, b, c, etc.) denote statistically significant differences (*p* < 0.05).

Compared to the control, the chlorophyll fluorescence intensity of both wild-type tobacco and *AfZFP5* transgenic tobacco significantly decreased under NaCl and NaHCO_3_ stress. However, the intensity remained higher in *AfZFP5* transgenic tobacco than in wild-type tobacco. This indicates that *AfZFP5* transgenic tobacco has a stronger ability to maintain photosynthesis under salt–alkali stress, demonstrating greater tolerance to these conditions (Figure 12A).

Under NaCl and NaHCO_3_ stress, the expression levels of SOD and POD marker genes in *AfZFP5* transgenic tobacco were significantly upregulated compared to wild-type tobacco. Under NaCl treatment, the expression level of SOD marker gene in the T3-3 strain was upregulated approximately 15-fold, while in T3-4 and T3-5 strains, it was upregulated about 10-fold. Similarly, the expression levels of the POD marker genes were upregulated approximately 2.5-fold. Under NaHCO_3_ treatment, the expression levels of SOD and POD marker genes were upregulated about 1.5-fold. These results suggest that the higher SOD and POD activities in *AfZFP5* transgenic tobacco under NaCl and NaHCO_3_ stress may be attributed to the elevated expression levels of SOD and POD marker genes in *AfZFP5* transgenic tobacco compared to wild-type tobacco (Figure 12B–E).

## 3. Discussion

Drought and soil salinization are the major constrains on crop production [29,30]. ZFPs play an important role in plant responses to abiotic stress [31]. In this study, the effects of various stress treatments on the expression levels of *AfZFP5* in the roots and leaves of *Amorpha fruticosa* were analyzed. Compared to the control, the expression level of *AfZFP5* in the roots and leaves significantly increased after 24 h of treatment with 100 mM sorbitol (Figure 4A,B). Under 1% H_2_O_2_ stress, the expression level of *AfZFP5* in the roots and leaves of *Amorpha fruticosa* exhibited unstable fluctuations (Figure 4C,D). Treatment with 100 mM NaCl significantly upregulated *AfZFP5* in both roots and leaves (Figure 4E,F). In contrast, 50 mM NaHCO_3_ stress significantly downregulated *AfZFP5* in the roots but upregulated it in the leaves, with the most pronounced effect observed at 12 h of treatment (Figure 4G,H). The differential expression of *AfZFP5* under various stress conditions suggests its potential involvement in multiple stress pathways. For example, OsLOL5 ZFP can regulate oxidative stress and respond to salt stress [32]. Meanwhile, subcellular localization analysis revealed that pBI121-*AfZFP5*-GFP fusion expression was located in the nucleus (Figure 5). Based on these results, it is hypothesized that *AfZFP5* may function in signal transduction pathways to defend against damage caused by abiotic stress.

*AfZFP5* transgenic tobacco exhibits enhanced tolerance to drought and salt–alkali stress during germination, seedling growth, and the vegetative stage. The germination rates, root lengths, and fresh weights of *AfZFP5* transgenic tobacco treated with sorbitol, NaCl, and NaHCO_3_ were significantly higher than those of wild-type tobacco (Figure 6 and Figure 7). Notably, treatments with 125 mM and 150 mM NaCl significantly promoted root elongation in *AfZFP5* transgenic tobacco. However, the mechanism by which these specific NaCl concentrations enhance root growth in transgenic tobacco requires further investigation (Figure 7I,J). Additionally, POD activity in *AfZFP5* transgenic tobacco at the seedling stage was significantly stronger than in wild-type tobacco under sorbitol and NaCl treatments. This suggests that the stronger antioxidant activity of transgenic tobacco contributes to increased tolerance to stress (Figure 7D–L). While H_2_O_2_ treatment did not inhibit germination, it did suppress growth. Nevertheless, the fresh weight, root length, and chlorophyll content of *AfZFP5* transgenic tobacco during the seedling stage were significantly better than those of wild-type tobacco under H_2_O_2_ stress (Figure 6 and Figure 7E–H). These findings further confirm that transgenic tobacco protects its cells from oxidative damage through the synergistic effect of its antioxidant defense system.

Under natural drought, NaCl, and NaHCO_3_ stress treatments, the activities of SOD and POD enzymes in *AfZFP5* transgenic tobacco during the vegetative stage were significantly increased (Figure 9B,C, Figure 10D,E and Figure 11D,E). Under NaCl and NaHCO_3_ stress, *AfZFP5* transgenic tobacco exhibited lighter DAB and NBT staining and significantly lower MDA content compared to wild-type tobacco (Figure 10B,C and Figure 11B,C). These results indicate that *AfZFP5* transgenic tobacco experiences less membrane lipid peroxidation and accumulates lower levels of ROS under stress compared to wild-type tobacco. qRT-PCR analysis revealed that the expression levels of NtSOD and NtPOD in *AfZFP5* transgenic tobacco were significantly higher than those in wild-type tobacco (Figure 9D,E and Figure 12B–E). This suggests that *AfZFP5* may enhance tolerance to drought and salt–alkali stress in transgenic plants by reducing ROS accumulation and increasing SOD and POD enzyme activities. Furthermore, analysis of photosynthetic characteristics under simulated drought with PEG6000, NaCl, and NaHCO_3_ stress treatments showed that the chlorophyll fluorescence intensity and Fv/Fm ratio of *AfZFP5* transgenic tobacco were significantly higher than those of wild-type tobacco (Figure 8A and Figure 12A). This indicates that *AfZFP5* plays an important role in mitigating damage to the photosynthetic system under stress [33].

## 4. Materials and Methods

### 4.1. Plant Material

Seeds of *Amorpha fruticosa* were collected from the East Road Green Belt in Ning jiang District, Song Yuan City, Jilin Province, China. Tobacco seeds were preserved by the Key Laboratory of Saline Alkali Land Vegetation Restoration and Reconstruction, Ministry of Education, Northeast Forestry University.

### 4.2. Strain, Vector, and Reagents

*E. coli* JM109 and *Agrobacterium* EHA105 strains were stored in our laboratory. The pMD18-T vector was purchased from TaKaRa. The pBI121-MCS-GFP plant expression vector was stored in our laboratory. T4-DNA ligase and Ex-Taq DNA polymerase were purchased from TaKaRa. The real-time quantitative fluorescent dye SYBR Green qPCR Master Mix and Gel Extraction Kit was purchased from respective suppliers. Acetosyringone was purchased from Solarbio Biologicals. RNA extraction kits, gel recovery kits, and plasmid extraction kits were purchased from Kangwei Company (Beijing, China). Other reagents were sourced from Yongda chemical reagent Co., Ltd. (Tianjin, China).

### 4.3. Cloning and Bioinformatics Analysis of AfZFP5

Specific primers (AfZFP5-F1:ATGGAAAAGGATACATATGTCTGTG and AfZFP5-R1: TTACGTCCTCCTTCGAGAGCT) were designed using Primer 5.0 software. The full-length sequence of *AfZFP5* was amplified by RT-PCR and subsequently ligated into the pMD18-T vector. The recombinant vector was then transformed into the *E. coli* TOP10 component via heat shock. PCR screening was performed on the bacterial culture, and after successful identification, plasmid extraction was performed using the Beijing Kangwei Century Plasmid Extraction Kit (Beijing, China). The re combinant plasmid was shipped to Kumei Company for sequencing.

The *AfZFP5* sequence was analyzed using the NCBI online tool. The primary structure of AfZFP5 was predicted using The SMART (SMART: Main page (embl-heidelberg.de)), while its secondary and tertiary structures were predicted using SWISS-MODEL. Phylogenetic analysis was performed using MEFA7.0.

### 4.4. Expression Characteristics of AfZFP5

Total RNA was extracted from various tissues (roots, stems, leaves, and flowers) of healthy perennial *Amorpha fruticosa*. Four-week-old *Amorpha fruticosa* seedlings were subjected to stress treatments using 100 mM sorbitol, 1% H_2_O_2_, 100 mM NaCl, and 50 mM NaHCO_3_. Leaves and roots were sampled at 0, 6, 12, 24, and 48 h after each treatment, and total RNA was extracted. cDNA was synthesized through reverse transcription and used as a template for qRT-PCR. Specific primers (AfZFP5-F2: GCCAGGAAGGCTAGCATGAA and AfZFP5-R2: ATATGCCCCTGGTTTGAGGC) for the internal reference and target gene (primers-AfTubu-F: ACAAGGCGGTTAAGGTTGGT and AfTubu-R: GTTCTGGGCTTGGTTCCCTT) were designed for quantification reactions. Data were collected using the MxPro-Mx3000P system, analyzed using the IBM SPSS Statistics 27 data processing system, and visualized using Origin software.

### 4.5. Subcellular Localization Analysis of AfZFP5

Specific primers (AfZFP5-F3: TCTAGATATGGAAAAGGATACATAT and AfZFP5-R3: GTCGACTCGTCCTCCTTCGAGAGCT) were designed by introducing XbalI and SalI restriction sites. Using pMD18-T-*AfZFP5* as a template, PCR amplification was performed on the ORF region of *AfZFP5*, followed by gel recovery. pBI121-MCS-GFP was double-digested and ligated with the gel recovery product to obtain the recombinant plasmid pBI121-*AfZFP5*-GFP (a binary plant expression vector under the control of the CaMV 35S promoter). *Agrobacterium tumefaciens* EHA105 containing the pBI121- *AfZFP5*-GFP recombinant plasmid was selected for injection into fresh tobacco leaves. The infiltrated tobacco plants were incubated in the dark for 12–16 h and then transferred to normal light for 3 days. Subcellular localization was observed using confocal microscopy, with fluorescence and DAPI staining used to confirm localization.

### 4.6. Acquisition of Transgenic Tobacco

Tobacco leaves were infected with recombinant *Agrobacterium tumefaciens* EHA105 containing pBI121-*AfZFP5*-GFP plasmid DNA and cultured on MS As medium for 3 days. Bud differentiation was induced on tobacco screening differentiation medium supplemented with 50 mg/L Kanamycin. Rooting was further induced on rooting medium containing 1/2 MS, 50 mg/L Kana, and 250 mg/L carbenicillin. qRT-PCR analysis was performed on T1 transgenic tobacco and wild-type tobacco plants to detect relative gene expression levels. Three high-expression transgenic lines were selected for subsequent experiments. T1 generation seeds were harvested from pot cultures and T3 generation seeds were obtained through Kanamycin screening.

### 4.7. Analysis of Drought and Salt–Alkali Stress Resistance in Tobacco Overexpressing AfZFP5

T3-5 strain *AfZFP5* transgenic tobacco seeds were transplanted into 1/2 MS medium supplemented with sorbitol (100 mM, 200 mM, 300 mM), H_2_O_2_ (2 mM, 3 mM, 4 mM), NaCl (125 mM, 150 mM, 175 mM), and NaHCO_3_ (2.5 mM, 5 mM, 7.5 mM). The pre-seeded medium was placed horizontally in an artificial constant-temperature climate chamber at 25 °C with a 16 h light/8 h dark photoperiod. During the experiment, the number of germinated seeds was recorded simultaneously. Seed germination was defined as the embryo root breaking through the seed coat by 1 mm. Germination was considered complete when no new seeds germinated for four consecutive days, and the germination rate was calculated.

Wild-type and *AfZFP5* transgenic tobacco seeds were evenly sown and vertically cultured in 1/2 MS medium without any stress treatment. After seven days of germination, tobacco seedlings were transferred to 1/2 MS medium supplemented with sorbitol (100 mM, 200 mM, 300 mM), H_2_O_2_ (2 mM, 3 mM, 4 mM), NaCl (125 mM, 150 mM, 175 mM), and NaHCO_3_ (2 mM, 4 mM, 6 mM) for vertical cultivation. When significant phenotypic differences were observed between wild-type and *AfZFP5* transgenic tobacco seedlings, statistical analyses were conducted on root length and fresh weight. Relevant physiological indicators were measured.

Wild-type and *AfZFP5* transgenic tobacco seeds were sown in soil and cultivated under light exposure at 25 °C with an 8/16 h light/dark cycle. Natural drought stress, PEG6000=simulated drought stress, and salt–alkali stress experiments were conducted on wild-type and *AfZFP5* transgenic tobacco during their vegetative stage. The phenotype was recorded and SOD activity, POD activity, MDA content, and chlorophyll fluorescence (using FluorCam open chlorophyll fluorescence imaging system) were measured [34]. DAB and NBT staining was used to detect superoxide anions in tobacco leaves [35]. RNA samples were collected from both experimental and control groups, and NtActin was used as an internal reference to analyze the expression levels of oxidative stress-related genes.

## 5. Conclusions

In summary, this study reveals the regulatory role of *AfZFP5* in response to drought and salt–alkali stress through mechanisms involving ROS balance and repair of photosynthetic system damage. These findings provide a foundation for further research into how *AfZFP5* enhances plant tolerance to abiotic stress and offer valuable insights for the development of drought-resistant and salt–alkali-tolerant crop breeding strategies.

## Data Availability

This article does not contain any content that needs to be uploaded to the database.

## Supplementary Materials

The following supporting information can be downloaded at https://www.mdpi.com/article/10.3390/ijms26083792/s1.
