# Supplementary material for: Cloning and Functional Analysis of ZFP5 from Amorpha fruticosa for Enhancing Drought and Saline–Alkali Resistance in Tobacco
Source: Int J Mol Sci. 2025 Apr 17;26(8):3792. doi: 10.3390/ijms26083792 (PMC12028205; doi:10.3390/ijms26083792)
Supplement: Supplementary file 1 [file ijms-26-03792-s001.zip › ijms-3558470-Supplementary.pdf]

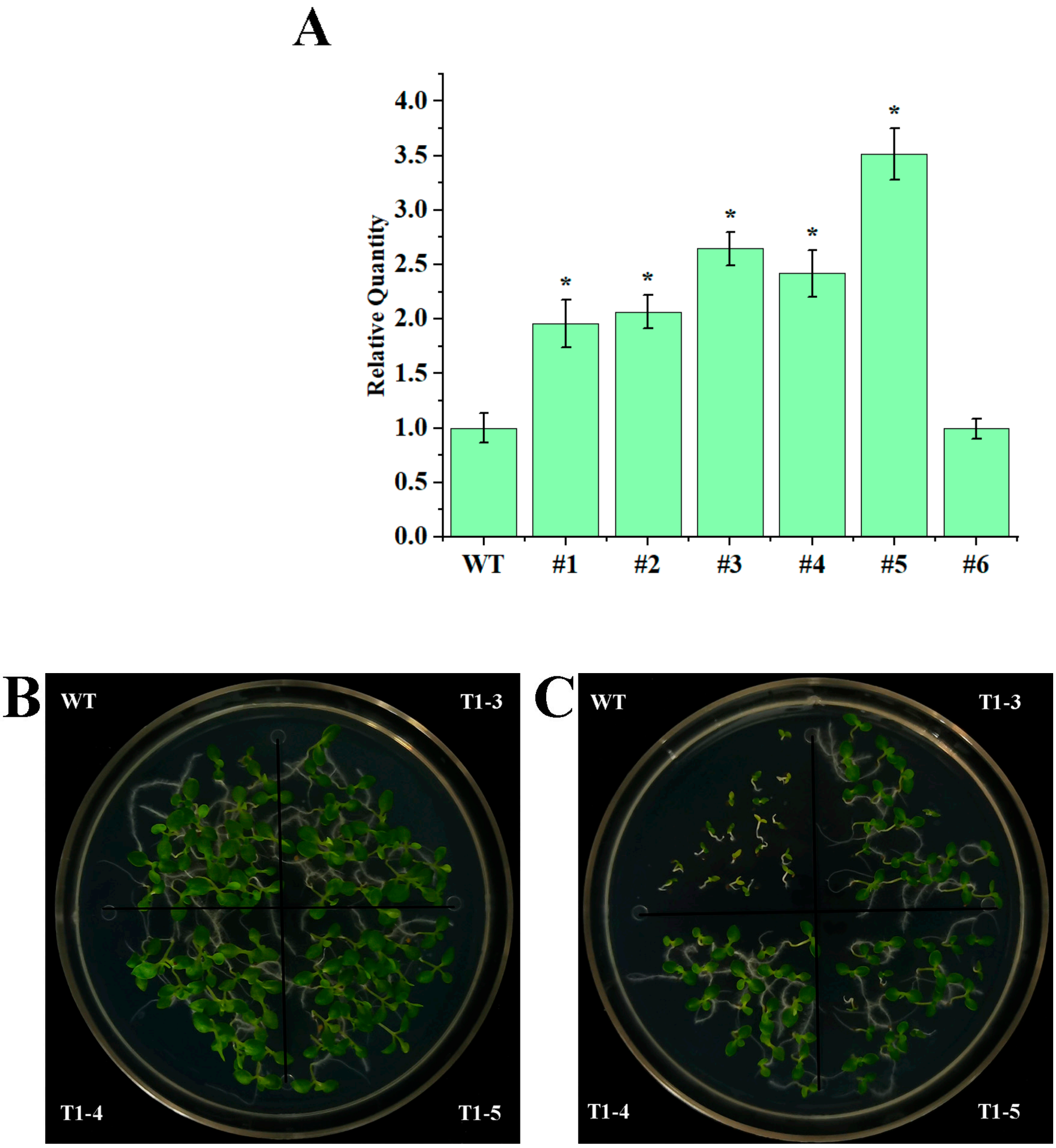

Supplementary Figure S1. Overexpression of *AfZFP5* in tobacco. (A) qRT-PCR analysis of AfZFP5 expression in six transgenic lines. (B)-(C) Kanamycin resistance screening of transgenic tobacco plants. Note: The error bars represent the standard errors of three biological replicates. Significant differences were determined at $p<0.05$ level.

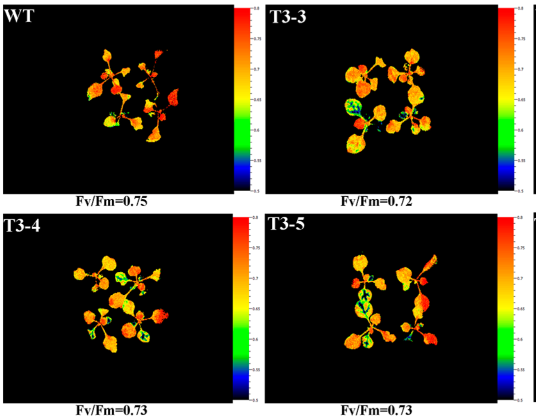

Supplementary Figure S2. Chlorophyll fluorescence imaging of *AfZFP5* transgenic tobacco during the vegetative stage before PEG6000-simulates drought treatment.
